# Reply to Takada and Komine-Aizawa, “Bacterial vaginosis and the gut-vagina axis”

**DOI:** 10.1128/spectrum.01801-25

**Published:** 2026-03-17

**Authors:** Mo Li, Qianyu Zhang, Tong Wu, Dianxing Hu, Shixuan Wang, Jinjin Zhang

**Affiliations:** 1 Department of Obstetrics and Gynecology, Tongji Hospital, Tongji Medical College, Huazhong University of Science and Technology 12443, Wuhan, Hubei, China; 2 National Clinical Research Center for Obstetrical and Gynecological Diseases, Wuhan, Hubei, China; Chengdu University, Chengdu, Sichuan, China

## REPLY

Thank you for your thoughtful commentary. We fully agree that “vaginitis” is an umbrella term that comprises several distinct clinical entities, most notably bacterial vaginosis (BV), vulvovaginal candidiasis, and trichomoniasis, and future investigations of the gut–vagina axis should therefore be stratified by etiology.

Our study was constrained by the available genome-wide association studies (GWAS) summary statistics (GCST90044303), which aggregate all ICD-10 codes mapped to PheCode 614.52 (N76.0–N76.3, N77.1, and A56.02). We now realize that this pooled phenotype obscures the heterogeneity you highlight. Although the majority of cases likely represent non-specific or mixed etiologies, we lacked the granular data to isolate BV-specific signals or to apply Amsel/Nugent criteria. Consequently, our reported associations between gut taxa (e.g., Candidatus Soleaferrea, Dialister, and Lachnospiraceae UCG-008) and “vaginitis” should be interpreted cautiously; they probably reflect shared microenvironmental perturbations, such as pH shifts or systemic immune modulation, rather than causal links to any single condition.

We are committed to addressing this limitation. As larger, etiology-specific GWAS and high-resolution multi-omics cohorts emerge, we plan to perform stratified Mendelian randomization analyses for BV, candidiasis, and trichomoniasis separately. Such efforts will be essential to disentangle the distinct microbial and metabolic pathways that traverse the gut–vagina axis.

We appreciate your constructive critique and look forward to the field moving toward the precision you advocate.

